# Single Subcutaneous Nodule as Initial Presentation of Atypical Lung Carcinoid

**DOI:** 10.4021/wjon246w

**Published:** 2010-11-02

**Authors:** Run Yu, Edward Wolin, Xuemo Fan

**Affiliations:** a Carcinoid and Neuroendocrine Tumor Center, Cedars-Sinai Medical Center, Los Angeles, California, USA; b Department of Pathology, Cedars-Sinai Medical Center, Los Angeles, California, USA

**Keywords:** Subcutaneous nodule, Atypical lung carcinoid, Metastasis

## Abstract

We report a 50-year-old woman whose atypical lung carcinoid presented as a single subcutaneous nodule. Biopsy of the nodule revealed neuroendocrine carcinoma likely from the lungs. Imaging studies revealed masses in lungs, pelvis and right axilla, and a seizure a few months later lead to discovery of brain metastasis. This case illustrates that an apparently innocuous subcutaneous nodule could be the initial presentation of an otherwise asymptomatic but widely metastatic atypical lung carcinoid. Physicians should be aware of signs of skin metastasis from lung malignancies and judiciously select patients with subcutaneous nodule for biopsy.

## Introduction

Subcutaneous nodules are common clinical findings and mostly benign lesions; they can also be metastatic cancer from an internal organ [1]. Atypical lung carcinoid is an intermediate-grade neuroendocrine carcinoma which usually presents with shortness of breath, hemoptysis, or as an incidental finding [2]. We describe here a single subcutaneous nodule as the initial presentation of atypical lung carcinoid.

## Case Report

**Figure 1:**
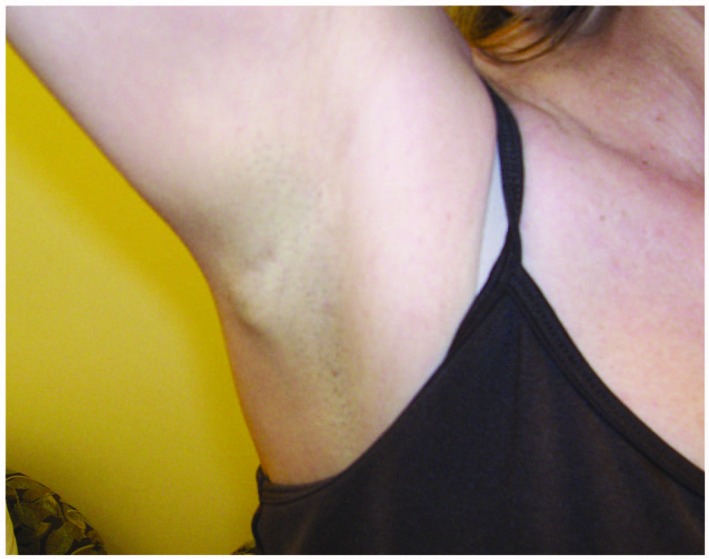
The subcutaneous nodule in the patient’s right axilla.

**Figure 2:**
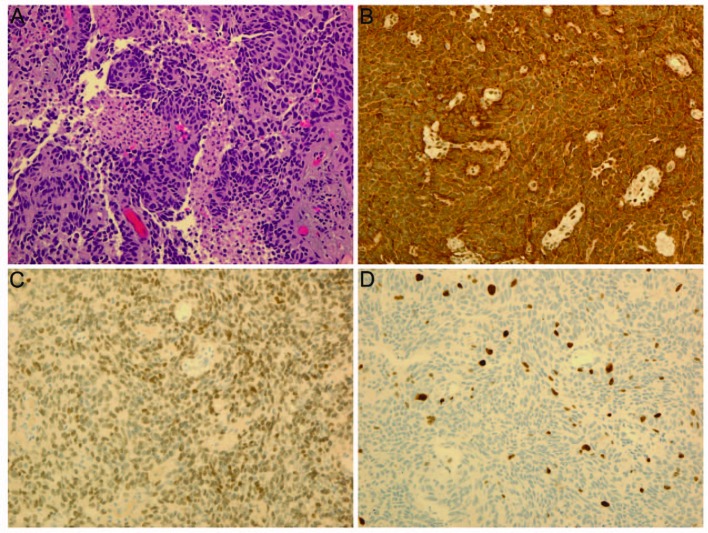
Pathology of biopsy samples from the subcutaneous nodule: (A) Hematoxylin and eosin staining; (B) Immunostaining of chromogranin A; (C) Immunostaining of thyroid transcription factor-1, a lung marker; (D) Ki-67 labeling (5-10%).

**Figure 3:**
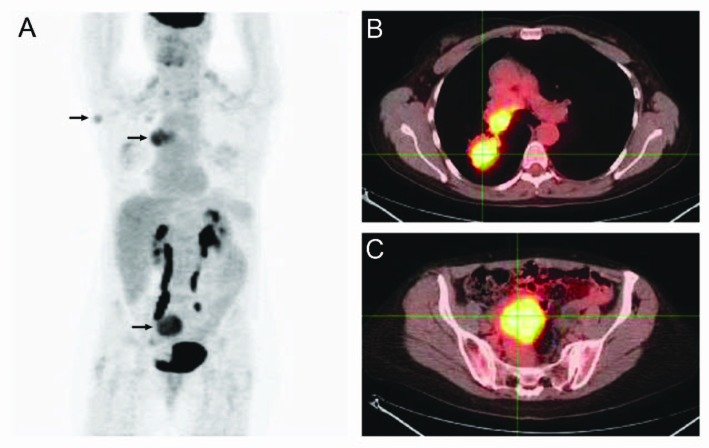
PET/CT study: (A) Whole body PET; (B) PET/CT overlay of chest; (C) PET/CT overly of pelvis. Arrows: hypermetabolic foci on PET.

A 50-year-old woman presented with a single subcutaneous nodule in her right axilla. The patient first felt the nodule 1.5 years before, but ignored it as she thought it might be part of fibroadenomatous breast disease. The mass rapidly grew to 2.1 cm and became harder in the previous few months (Fig. 1). The patient otherwise felt well. She noted significant weight loss of 35 pounds during the same period which she contributed to voluntary diet and exercise and she felt more energetic after the weight loss. She had a 25-pack-year history of smoking. Multiple cancers were reported in the patient’s family members. Breast ultrasound and mammogram revealed bilateral benign-appearing cysts unchanged from those found in previous studies. Ultrasound showed high vascularity in the right axillary mass which was biopsied. Pathological examination demonstrated that the tumor consisted of fascicles of spindle cells with small areas of necrosis, mild cytologic atypia, and a low mitotic rate. Immunostaining showed strong positivity for chromogranin and synaptophysin and moderate positivity for TTF-1 (thyroid transcription factor-1), a lung marker (Fig. 2). Breast cancer markers were negative. Ki-67 labeling index was 5-10%. These findings were consistent with an intermediate-grade neuroendocrine carcinoma probably from the lungs. Chest CT showed two right hilar masses, measuring 4.3 x 3.5 cm and 3.1 x 2.9 cm, right hilar and pretracheal adenopathy, and a 1.8 cm right middle lobe solitary lung nodule. These lung masses were hypermetabolic on PET/CT which also revealed a 4.9 x 4.4 cm hypermetabolic mass in the pelvis and identified the right axiallary nodule as mildly hypermetabolic (Fig. 3). No liver metastasis was noted. Chromagranin A and neuron-specific enolase levels were normal. Pancreastatin levels were 192 pg/ml (10 - 135), ACTH 85 pg/ml (6 - 58), serotonin 234 ng/ml (22 - 180), and cortisol 17.8 mcg/dl (3 - 14). Octreotide scan failed to detect any foci. The patient was diagnosed with metastatic atypical lung carcinoid and chemotherapy was planned. Before initiating chemotherapy, she had a brief episode of altered mental status that was consistent with seizure. Brain MRI demonstrated a left temporal lobe metastasis which was surgically resected. Pathological examination of surgical samples revealed similar features to those in the subcutaneous nodule.

## Discussion

In this report, we describe a single subcutaneous nodule as the initial presentation of atypical lung carcinoid. Skin involvement from internal cancers is rare and is even rarer as their initial presentation [1]. About 2% of patients with lung carcinoma eventually develop skin metastasis and only 0.3% present with skin involvement. In patients with carcinoids, the percentage of those with skin involvement as initial presentation is not known, but likely very small [3].

Atypical lung carcinoids are intermediate-grade neuroendocrine tumors of the lungs with a five-year survival rate of around 70% [2]. They are usually found in middle-aged adults with a smoking history. Patients with this disease usually exhibit coughing, wheezing, hemoptysis, or weight loss. When large air ways are blocked, patients may develop post-obstructive pneumonia. The tumors sometimes produce ACTH, leading to hypercortisolemia, as in our patient. Atypical lung carcinoids metastasize at a higher rate than typical lung carcinoids, but lower than small cell lung cancer, and the most common site of metastasis is the liver. Skin metastasis from atypical lung carcinoids is rare. To our knowledge, there are only a couple of cases besides ours in the English literature where atypical lung carcinoid metastasizes to the skin [4-6]. In those cases, the atypical lung carcinoids present with multiple metastatic skin nodules that are often painful and erythematous, suggesting malignancy. Our patient is the only one that presents with a single subcutaneous nodule which initially appears harmless.

Skin nodules are a common clinical finding, and most are benign. Therefore, when a skin nodule is discovered, several factors must be considered before it is suspected of being a metastasis. The nodule must be persistent. The patient’s family history and past medical history of malignancy, and other cancer risk factors, such as smoking, should raise the clinical suspicion of metastasis from lung malignancy. Rapid growth of skin nodules is an alarming sign of metastasis. The significant weight loss in our patient may also be a sign of malignancy. Timely biopsy of the nodule is important in establishing diagnosis and initiating therapy. In our patient, the history of atypical lung carcinoid immediately raised the concern of brain metastasis when she had the brief episode of altered mental status, leading to prompt diagnosis and definitive therapy of the brain metastasis.

In summary, our case illustrates how a seemingly innocuous subcutaneous nodule leads to the diagnosis of widely metastatic atypical lung carcinoid. Clinicians should be aware of signs of skin metastasis from lung malignancies and judiciously select those patients with significant clinical suspicion of malignancy to undergo biopsy.
